# Phosphatase of regenerating liver-3 (PRL-3) is overexpressed in classical Hodgkin lymphoma and promotes survival and migration

**DOI:** 10.1186/s40164-018-0100-2

**Published:** 2018-04-10

**Authors:** Magnus Aassved Hjort, Håkon Hov, Pegah Abdollahi, Esten Nymoen Vandsemb, Unn-Merete Fagerli, Bendik Lund, Tobias Schmidt Slørdahl, Magne Børset, Torstein Baade Rø

**Affiliations:** 1 Department of Clinical and Molecular Medicine, Faculty of Medicine and Health Sciences, Norwegian University of Science and Technology, (NTNU), P.O. Box 8905, 7491 Trondheim, Norway; 2 Children’s Clinic, Trondheim University Hospital, Trondheim, Norway; 3 Department of Pathology, Trondheim University Hospital, Trondheim, Norway; 4 Cancer Clinic, Trondheim University Hospital, Trondheim, Norway; 5 Department of Hematology, Trondheim University Hospital, Trondheim, Norway; 6 Department of Immunology and Transfusion Medicine St. Olavs Hospital, Trondheim University Hospital, Trondheim, Norway

**Keywords:** PRL-3, Hodgkin lymphoma, IL-13, STAT6, Migration, Survival

## Abstract

**Background:**

Phosphatase of regenerating liver-3 (PRL-3) is implicated in oncogenesis of hematological and solid cancers. PRL-3 expression increases metastatic potential, invasiveness and is associated with poor prognosis. With this study, we aimed to show a possible oncogenic role of PRL-3 in classical Hodgkin lymphoma (cHL).

**Methods:**

PRL-3 expression was measured in 25 cHL patients by immunohistochemistry and gene expression was analyzed from microdissected malignant cells. We knocked down PRL-3 in the cHL cell lines L1236 and HDLM2 and used small molecular inhibitors against PRL-3 to investigate proliferation, migration and cytokine production.

**Results:**

PRL-3 protein was expressed in 16% of patient samples. In three different gene expression datasets, PRL-3 was significantly overexpressed compared to normal controls. PRL-3 knockdown reduced proliferation, viability and Mcl-1 expression in L1236, but not in HDLM2 cells. Thienopyridone, a small molecule inhibitor of PRL-3, reduced proliferation of both L1236 and HDLM2. PRL-3 affected IL-13 secretion and enhanced STAT6 signaling. IL-13 stimulation partially rescued proliferation in L1236 cells after knockdown of PRL-3. PRL-3 knockdown reduced migration in both L1236 and HDLM2 cells.

**Conclusion:**

PRL-3 was overexpressed in a subset of cHL patients. Inhibition of PRL-3 increased IL-13 cytokine production and reduced migration, proliferation and viability. The effects could be mediated through regulation of the anti-apoptotic molecule Mcl-1 and a feedback loop of IL-13 mediated activation of STAT6. This point to a role for PRL-3 in the pathogenesis of Hodgkin lymphoma, and PRL-3 could be a possible new drug target.

**Electronic supplementary material:**

The online version of this article (10.1186/s40164-018-0100-2) contains supplementary material, which is available to authorized users.

## Background

Lymphoma is divided in Hodgkin lymphoma (HL) and non-Hodgkin lymphoma (NHL), with further subclassification based on immunophenotype, morphological, genetic and clinical features [1]. Lymphoma is the third most common childhood malignancy, and constitutes approximately 10% of all pediatric and 5% of adults cancers [2]. HL is for most cases a curable cancer, with 5-year survival rates of 98% in children < 15 years of age and 86% in adults [3, 4]. Still, these patients can suffer from reduced life expectancy because of increased risk for secondary malignancies and toxicity from treatment [5]. Thus, the challenge of HL is to find an optimal strategy for maintaining high cure rates and overall survival while simultaneously avoiding long-term side effects and risk for secondary malignancies associated with HL.

The hallmark of classical HL (cHL) is the malignant Hodgkin and Reed–Sternberg (HRS) cells. The HRS cells are derived from mature B cells, but they have a phenotype and expression of hematopoietic surface markers that do not resemble those of any other hematopoietic cells [6]. Several signaling pathways are deregulated in cHL, including constitutively active nuclear factor-κB (NF-κB) and Janus kinase-signal transducer and activator of transcription (JAK-STAT) signaling pathways. A salient feature in cHL is that the malignant cells often constitute only 0.1–2% of the tumor mass. The rest is composed of non-malignant inflammatory cells like T cells, B cells, plasma cells, macrophages, natural killer (NK) cells, dendritic cells, neutrophils, eosinophils, mast cells, and fibroblasts [6]. HRS cells secrete proliferative cytokines, like interleukin-6 (IL-6) and IL-13, which act in an autocrine and paracrine manner. The secretion of cytokines and interplay with the microenvironment, are crucial for survival of the malignant HRS cells [7–9].

We have previously found that phosphatase of regenerating liver-3 (*PTP4A3/PRL*-*3*) has oncogenic properties in multiple myeloma (MM) [10, 11] and acute lymphoblastic leukemia (ALL) [12]. PRL-3 is up-regulated in several solid and hematological cancers, and is associated with metastasis, survival and poor prognosis [13, 14]. Little is known about PRL-3 in lymphoma, except for high mRNA-expression in some cell lines [10, 15]. No PRL-3 protein expression is found in mature healthy tissues. This seemingly restricted expression to cancerous tissues, makes PRL-3 a tempting target for cancer treatment.

With this paper, we aimed to search for PRL-3 expression in cHL and elucidate a possible survival and migratory role of PRL-3 in cHL.

## Methods

### Cell lines

The cell lines HDLM2 (ACC 17), L1236 (ACC 530), SUP-B15 (ACC 389), MHH-CALL-4 (ACC 337), CRO-AP5 (ACC 215) were from DSMZ (Brauschweig, Germany). Reh was from ATCC (Rockville, MD, USA). OCI-Ly3, OCI-Ly10, SU-DHL-4 and Karpas 422 were a kind gift from Ph.D. June H. Myklebust at the Norwegian Radium Hospital, Oslo University Hospital (Oslo, Norway). Cells were cultured in RPMI-1640 and 10–20% heat-inactivated fetal calf serum, according to supplier’s recommendation, at 37 °C in a humidified atmosphere with 5% CO_2_. Growth media were replenished twice weekly. Peripheral blood mononuclear cells (PBMC) was isolated from healthy volunteers using density gradient centrifugation.

### Antibodies, cytokines and other reagents

Antibodies against PRL-3 (sc-130355), Myeloid cell leukemia-1 (Mcl-1) (sc-819), IL-13Rα1 (sc-398831) and IL-13Rα2 (sc-134363) were bought from Santa Cruz Biotechnology (Dallas, Texas, USA). Antibodies against phosphorylated (p) STAT1 (#9167), total STAT1 (#14994), pSTAT6 (#9361), total STAT6 (#9362) and B cell lymphoma-extra large (Bcl-xL) (#2762) were from Cell Signaling Technology (Beverly, MA, USA). Antibodies against GAPDH and PRL-3 (ab52976) were from Abcam (Cambridge, United Kingdom). Recombinant human C-C motif chemokine ligand 19 (CCL19) and IL-13 were from Peprotech (Rocky Hill, NJ, USA). The plasmid pLKO (non-silencing control) and shRNA-pLKO against PRL-3 were a kind gift from dr. Jim Lambert (University of Colorado, Denver, CO, USA) [16]. Neutralizing antibodies against IL-6 (MAB2061) and IL-13 (MAB213) were from R&D Systems (Minneapolis, MN, USA). PRL-3 inhibitor I (#P0108) (5-[[5-Bromo-2-[(2-bromophenyl)methoxy]phenyl]methylene]-2-thioxo-4-thiazolidinone) was from Sigma-Aldrich (St. Louis, MO, USA), Analog 3 (#Z44389470) was from Enamine Store (Ukraine) and thienopyridone was a kind gift from Professor John S. Lazo (University of Virginia, Charlottesville, VA, USA).

### Immunohistochemistry (IHC) and gene expression datasets

Paraffin-embedded tissue blocks from cHL patients were cut in 4 μm sections. The sections were pretreated in Target Retrieval Solution (Dako K8004, Oslo, Norway) for 20 min at 97 °C. Subsequently, sections were washed and incubated with primary antibody against PRL-3 (ab52976, 1:1000) for 40 min. Labelled polymer HRP anti-Rabbit (Dako K4011) was used for developing the stain. Microscopic images were obtained with a Lumenera Infinity 2 camera and Infinity analyze software, release 6.2 (Lumenera Corporation, Ottawa, Ontario, Canada) using a Nikon eclipse Ci microscope (Nikon Gmbh, Düsseldorf, Germany) at 400× magnification. PRL-3 expression was graded in positive or negative expression. Positive expression was defined as uniform positive staining in tumor cells compared to background staining. PRL-3 expression in normal lymph node and prostate cancer was used as a negative and positive control, respectively (Additional file 1).

We searched the open gene expression profiling source Oncomine (http://www.oncomine.org) and PubMed to identify gene expression datasets comparing PRL-3 expression in HRS cells with normal control cells.

### Lentiviral transduction for PRL-3 knockdown

Stable PRL-3 knockdown in HDLM2 and L1236 was done as previously described using pLKO-shRNA against PRL-3 or pLKO (control plasmid) to establish PRL-3 knockdown (L1236 shPRL-3 and HDLM2 shPRL-3) and control (L1236 shCTRL and HDLM2 shCTRL) cells, respectively [12, 16]. Transfected cells were grown in medium containing 0.2 µg/mL puromycin for selection.

### Immunoblotting

Cells were treated as indicated, then pelleted, lysed and immunoblotted as previously described [17]. Images were acquired using LI-COR Odyssey Fc and analyzed with Image Studio Software (LI-COR, Lincoln, Nebraska). All experiments were conducted at least three times.

### RNA isolation, cDNA synthesis and quantitative real time PCR (qRT-PCR)

Isolation of RNA and cDNA synthesis were performed as previously described [18]. For relative quantification, we used the comparative 2^−ΔΔCT^-method, with GAPDH as endogenous reference. All samples were run in triplicate. The following primers were used: PTP4A3 (Hs02341135_m1), GAPDH (Hs99999905_m1) and IL-13 (Hs00174379_m1) (TaqMan, Applied Biosystems, ThermoFisher Scientific, Waltham, MA, USA). Analysis was performed using Step One Software 2.3 (Applied Biosystems).

### Cell proliferation and viability

Cell proliferation was measured by counting cells seeded into a 24-well plate on day 1, 4 and 7 using Coulter Counter Z1 (Bechman Coulter, Fullerton, CA) or Countess Cell Counter (ThermoFisher). Viability and apoptosis were evaluated using Annexin V-FITC binding and propidium iodide (PI) uptake (APOPTEST-FITC kit; Nexins Research, Kattendijke, The Netherlands), as previously described [12]. For L1236, PI negative or positive were used as measurement for viable or dead cells, respectively.

### Relative ATP measurement

Cells (1 × 10^4^) were seeded into 96-well clear bottom plates (Corning, Corning, NY, USA), with cytokine or inhibitor, as indicated. Cellular ATP production was estimated using CellTiter-Glo^®^ assay as previously described [11]. All conditions were run in triplicate, and experiments were performed three times.

### Migration assay

Cells (4 × 10^5^) were seeded into the upper chamber of a transwell culture plate with a polycarbonate membrane with pore size 5 µm (HDLM2) or 8 µm (L1236) (Corning). CCL19 (50 ng/mL) was used as a chemoattractant for 6 h (HDLM2) or 22 h (L1236). The cells that migrated through the membrane to the lower chamber, were counted using Coulter Counter Z1 (Bechman Coulter, Fullerton, CA). All conditions were performed in duplicate, and all experiments were performed three times.

### Cytokine secretion

Bio-Plex Pro Human Cytokine 27-plex Assay (Bio-Rad, Hercules, California, USA) was used according to the manufacturer’s instruction. Supernatants of cell cultures (5 × 10^5^ cells/mL) were harvested 24 h after medium exchange, and IL-6 (DY26) and IL-13 (DY2213) secretion were measured using quantitative DuoSet ELISA (R&D systems, Minneapolis, USA), according to the manufacturer’s instructions.

### Confocal microscopy

Cells were allowed to adhere to Poly-l-lysine (0.01%) and then fixed with 4% paraformaldehyde before they were permeabilized with PBS, 5% human serum and 0.5% saponin (Sigma-Aldrich). PRL-3 was detected with directly conjugated PRL-3-Alexa 488 antibody (St. Cruz Biotechnology), and the nuclei were stained with Hoechst 33342 (ThermoFisher Scientific). Confocal imaging was performed using a Leica SP8 STED 3D microscope (Wetzlar, Germany). Imaris (Bitplane, Zurich, Switzerland) was used for imaging editing.

### Statistics

Statistical significance was determined using Student’s t test in IBM SPSS Statistics version 24.

### Ethics

The use and storage of patient samples was approved by the Regional Ethics Committee (Approval #28/99, 2013/1594 and 2015/700-03).

## Results

### PRL-3 was expressed in cHL patients and cell lines

**Fig. 1:**
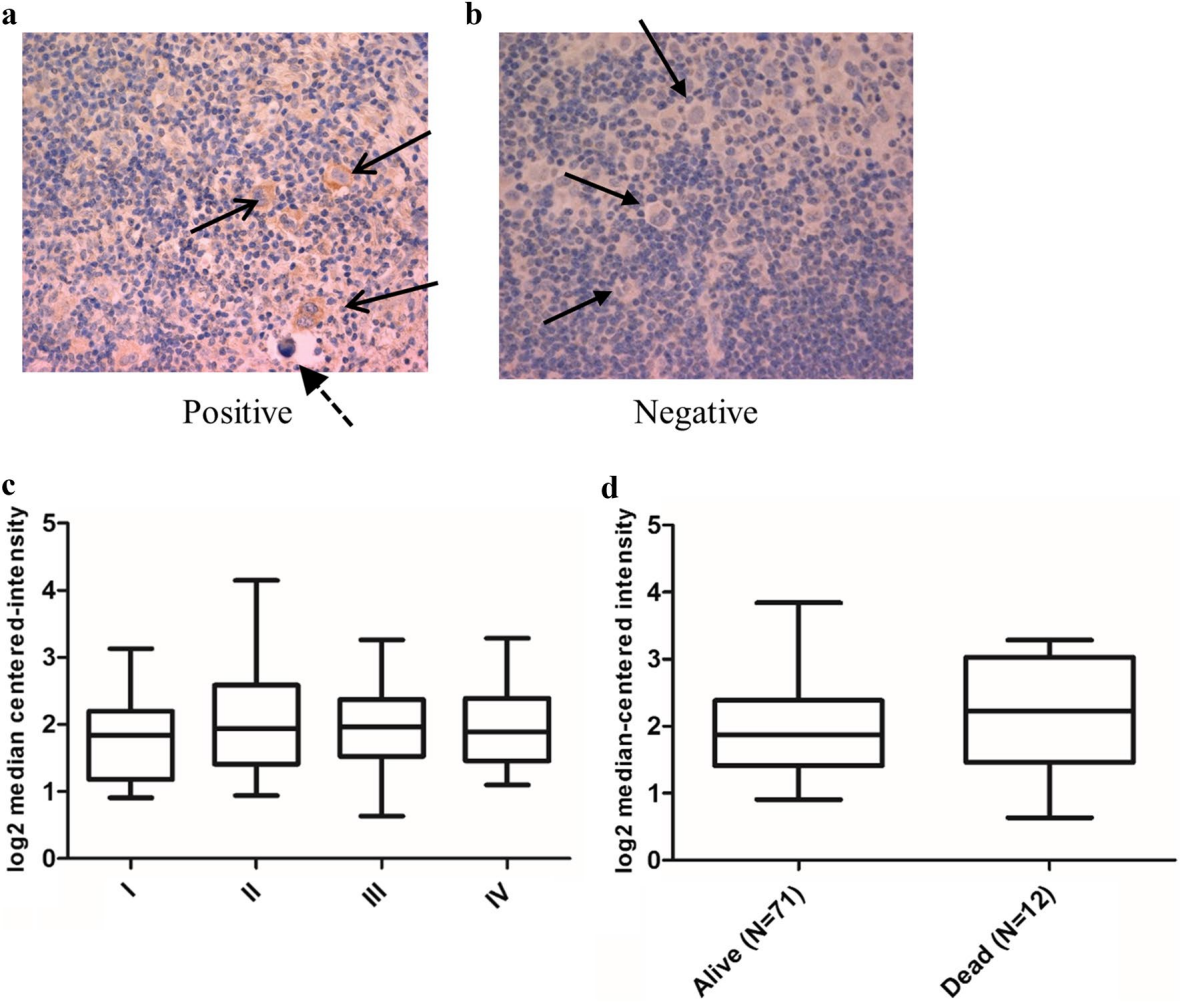
PRL-3 was expressed in cHL biopsies. **a** PRL-3 expression in HRS cells (arrows) and not in the surrounding cells measured by IHC. Dashed arrow indicates a so-called “mummy” cell. **b** IHC with negative PRL-3 expression in HRS cells (indicated by arrows). **c**, **d** *PRL*-*3* expression did not correlate to clinical endpoints like stage (**c**) or survival (**d**). Analysis of gene expression data from Steidl et al. [22]. Line in the middle of the box represent median and boxes represent 25–75 percentiles of log2 median-centered intensity of *PRL*-*3* expression. Whiskers represent minimum and maximum values (Data adapted from http://www.oncomine.org)

First, we examined PRL-3 protein expression in cHL patient samples. Lymph node biopsies from 25 patients were analyzed for PRL-3 expression by IHC. Four patients (16%) had positive PRL-3 expression, while 21 patients were classified as negative (84%) (Fig. 1a, b). We then analyzed *PRL*-*3* expression data from cHL samples compared to normal controls. Due to the low number of tumor cells in cHL, we searched for studies with expression data obtained from microdissected HRS cells. We identified three datasets comparing *PRL*-*3* expression in HRS cells to that in normal control cells [19–21]. *PRL*-*3* was significantly overexpressed in HRS cells in all three datasets (Table 1).

**Table 1:** PRL-3 expression in microdissected HRS cells compared to normal control

Dataset	Fold change	cHL sample number	Normal control number	p value	References
Brune et al.	2.19	12	25 (centroblast, B cells and follicle center cells)	0.002	[19]
Eckerle et al.	3.42	4	41 (NK and T cells)	0.013	[20]
Steidl et al.	11.5	29	5 (germinal center cells)	8.1 × 10^−10^	[21]

Table 1: PRL-3 was overexpressed in HRS cells compared to normal control cells in three different datasets. The malignant HRS cells constitute only 0.1–2% of the tumor volume. To obtain gene expression from only the HRS cells, all three datasets had isolated HRS cells by microdissection before gene expression was performed.

In our patient cohort, there was two deaths, and we did not find any difference in clinical factors such as stage, relapse or survival when comparing patients with positive or negative expression of PRL-3. We also analyzed data from Steidl et al. [22], and found no significant difference in stage (Fig. 1c), survival (Fig. 1d) or relapse (not shown), based on *PRL*-*3* expression.

**Fig. 2:**
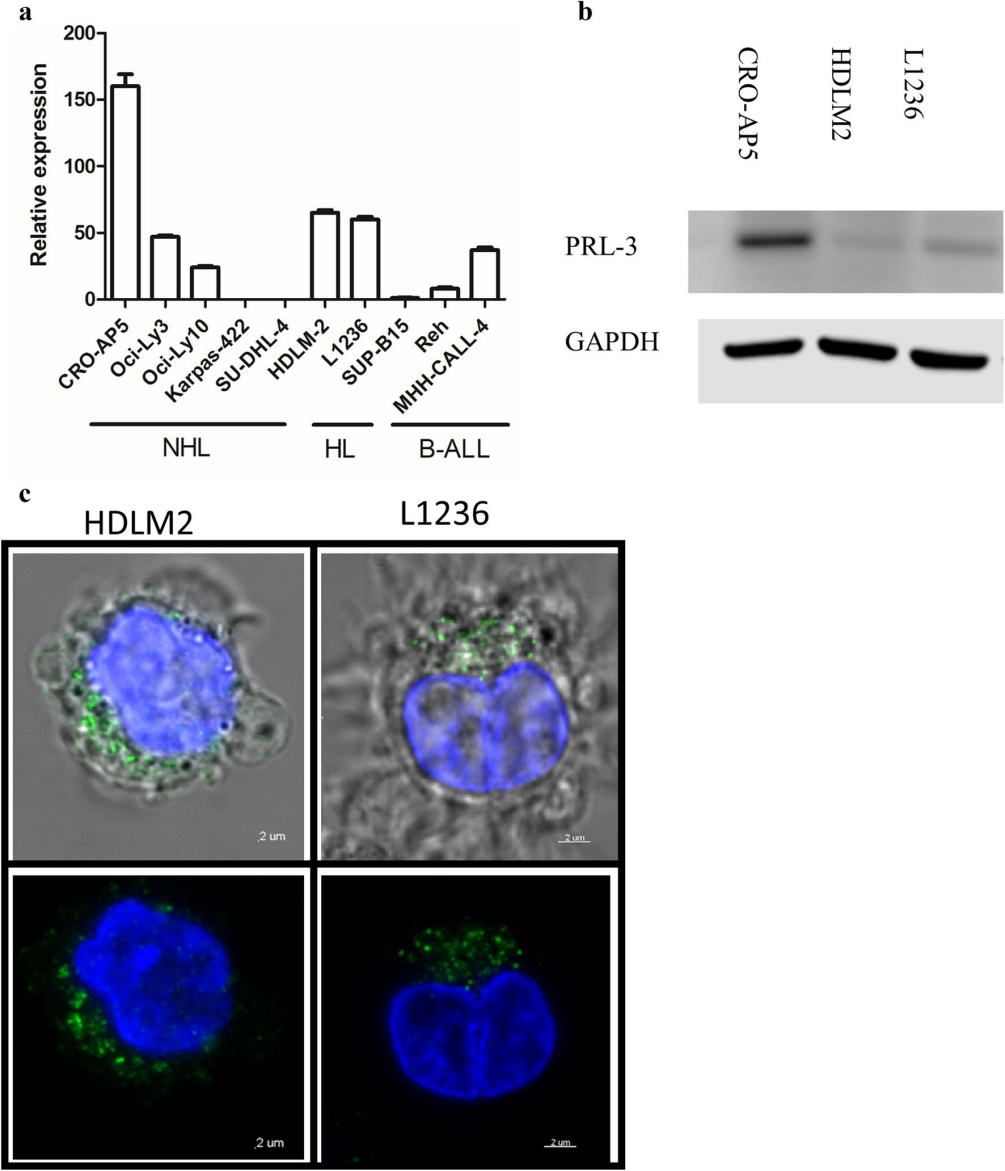
PRL-3 was expressed in cHL cell lines. **a** *PRL*-*3* expression in a selection of B cell cancer. Samples were normalized to their GAPDH level (2^−ΔΔCt^ method), and *PRL*-*3* expression in SUP-B15 was set to 1 [The ∆Ct between PRL-3 and GAPDH for SUP-B15 was 8.93 (± 0.2)]. Error bars represent + 1 SD of triplicates. **b**, **c** PRL-3 expression in HDLM2 and L1236 assessed by **b** western blot and **c** confocal microscopy. Green is anti-PRL-3 and blue is DNA (nucleus)

Next, we explored *PRL*-*3* expression B cell cancer cell lines. By qRT-PCR, we found high mRNA expression in L1236 and HDLM2, in three of the five examined B cell NHL cell lines and in three B cell ALL cell lines (Fig. 2a). The protein expression in HDLM2 was lower than in L1236, even though they had almost equal mRNA expression (Fig. 2b). By confocal microscopy, PRL-3 was detected in the cytoplasm of HDLM2 and L1236 (Fig. 2c).

### PRL-3 knockdown reduced survival of L1236

**Fig. 3:**
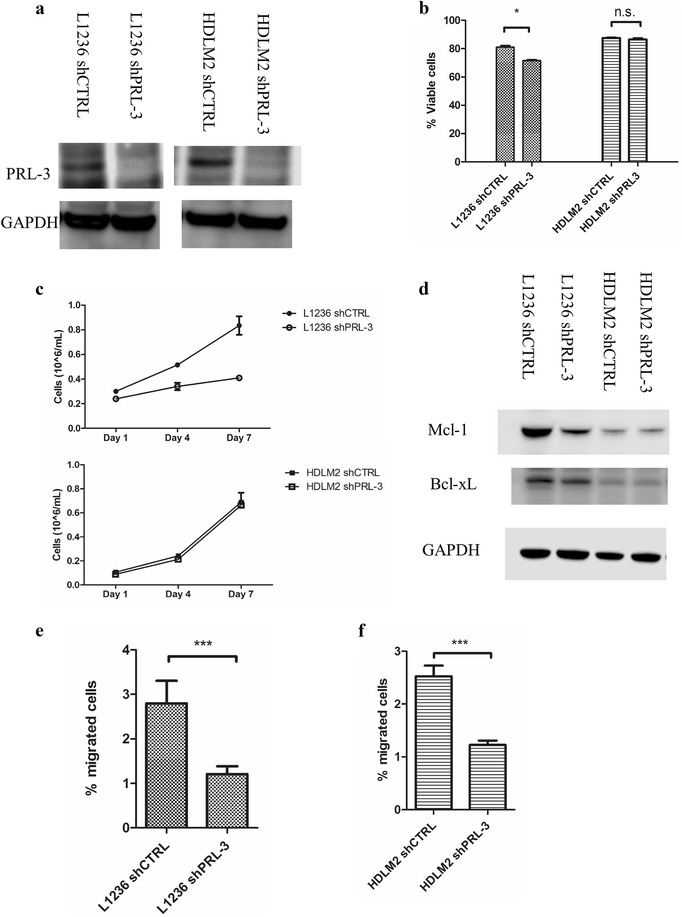
PRL-3 knockdown reduced survival and migration of cHL cells. **a** Western blot of PRL-3 knockdown by shRNA in L1236 and HDLM2. **b** Knockdown of PRL-3 reduced viability in L1236, but not in HDLM2, measured by annexin V-FITC/propidium iodide using flow cytometry. Bars represent percent viable cells from one one of three independent experiments with similar results. Error bars represent + 1 SD of duplicates. *p < 0.05. *ns* not significant. **c** PRL-3 knockdown reduced cell proliferation in L1236, but not in HDLM2 measured by cell counting. Error bars represent ± 1 SD of duplicates. Figure is showing one of three independent experiments with similar results. **d** Western blot of Mcl-1 and Bcl-xL expression. Membrane was reprobed for Bcl-xL and GAPDH. **e**, **f** Knockdown of PRL-3 reduced migration in **e** L1236 and **f** HDLM2. Bars represent the percentage of migrated cells from one of three independent experiments with similar results. Error bars represent + 1 SD of three repeated counts in two parallels. ***p < 0.001

To further examine a possible oncogenic role of PRL-3 in cHL, we examined survival and migration in cHL cells. To inhibit the activity of PRL-3, we created stable PRL-3 knockdown in L1236 (L1236 shPRL-3) and HDLM2 (HDLM2 shPRL-3), and control cells transfected with the empty vector pLKO (L1236 shCTRL and HDLM shCTRL). The knockdown of PRL-3 in both L1236 and HDLM2, as measured by qRT-PCR, was 85% efficient (data not shown) compared to controls and was confirmed at protein level (Fig. 3a).

In L1236 cells, knockdown of PRL-3 resulted in significantly reduced viability, but not in HDLM2 (Fig. 3b). Cell proliferation, as measured by cell counts on day 4 and 7, was reduced in L1236 shPRL-3 compared to L1236 shCTRL cells. HDLM2 shPRL-3 on the other hand, proliferated at the same rate as HDLM2 shCTRL (Fig. 3c). By western blotting, there was reduced amount of Mcl-1 protein in L1236 shPRL-3, but not in HDLM2 shPRL-3 (Fig. 3d). The amount of Bcl-xL, another important anti-apoptotic protein in cHL, was unchanged following knockdown of PRL-3 in L1236 and HDLM2. The reduced level of Mcl-1, points to a possible explanation for the reduced viability of L1236 cells after PRL-3 knockdown.

### PRL-3 knockdown reduced migration of cHL cells

In addition to cancer cell survival, PRL-3 has previously been associated with metastasis and cancer cell migration. The chemokine CCL19 is a known stimulator for migration of cHL cells. We therefore examined migration of cells with or without PRL-3 knockdown. In a transwell assay, CCL19 stimulated migration of both cell lines. Knockdown of PRL-3 reduced cell migration with 56 and 51% in L1236 and HDLM2, respectively (Fig. 3e, f).

### PRL-3 inhibitors reduced cell survival in cHL cells

**Fig. 4:**
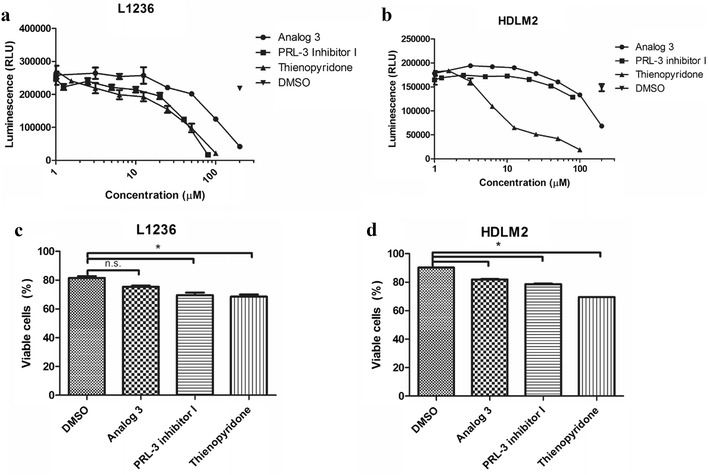
PRL-3 inhibitors reduced survival in cHL cell lines. **a**, **b** 48 h treatment with PRL-3 inhibitors reduced proliferation in **a** L1236 and **b** HDLM2 as measured by CellTiter-Glo^®^. Figure shows one representative of three independent experiments. Error bars represent ± 1 SD of triplicates. **c**, **d** Analog 3 (100 µM), PRL-3 inhibitor I (40 µM), and thienopyridone (10 µM) treatment for 48 h induced apoptosis in **c** L1236 and **d** HDLM2. Bars represent percent viable cells from one of three independent experiments with similar results. Error bars represent + 1 SD duplicates. Viability was determined by annexin V-FITC/propidium iodide using flow cytometry. *p < 0.05. *ns* not significant

To further explore the role of PRL-3 in the survival of cHL cells, we treated L1236 and HDLM2 with the three small molecular PRL-3 inhibitors Analog 3, PRL-3 inhibitor I and thienopyridone. We observed a dose-dependent reduction in proliferation of L1236 with all three inhibitors (Fig. 4a). HDLM2 cells were sensitive to thienopyridone and to some extent to Analog 3, in a dose-dependent manner, whereas they were resistant to PRL-3 inhibitor I (Fig. 4b). The IC_50_ values in L1236 for Analog 3, PRL-3 inhibitor I and thienopyridone were approximately 100, 40 and 30 µM, respectively. For HDLM2 the IC_50_ values for Analog 3 and thienopyridone were 180 and 7 µM, respectively. Analog 3 (100 µM) and PRL-3 inhibitor I (40 µM) did not reduce the proliferation of PBMC from two different healthy volunteers, while thienopyridone (25 µM) reduced the proliferation of PBMC from one of the donors (Additional file 2). All three inhibitors induced apoptosis in both cell lines, although not significantly for Analog 3 in L1236 compared to DMSO solvent control (Fig. 4c, d). Taken together, this indicates that PRL-3 inhibition can abrogate cHL cell survival.

### PRL-3 regulated cytokine production in cHL cells

A common feature of cHL cells is the secretion of distinct cytokines promoting proliferation and survival. We therefore used a multiplex assay to screen PRL-3 knockdown and control cells for differences in cytokine secretion. We found that IL-6, IL-9, IL-13, granulocyte–macrophage colony-stimulating factor (GM-CSF), C-X-C motif chemokine ligand 10 (CXCL10), CCL4, CCL5 and vascular endothelial growth factor (VEGF) were differentially secreted in PRL-3 knockdown compared to control in either L1236 and/or HDLM2 (data not shown). In subsequent experiments, we chose to focus on IL-6 and IL-13, as these cytokines previously have been linked to PRL-3 or cHL pathogenesis.

**Fig. 5:**
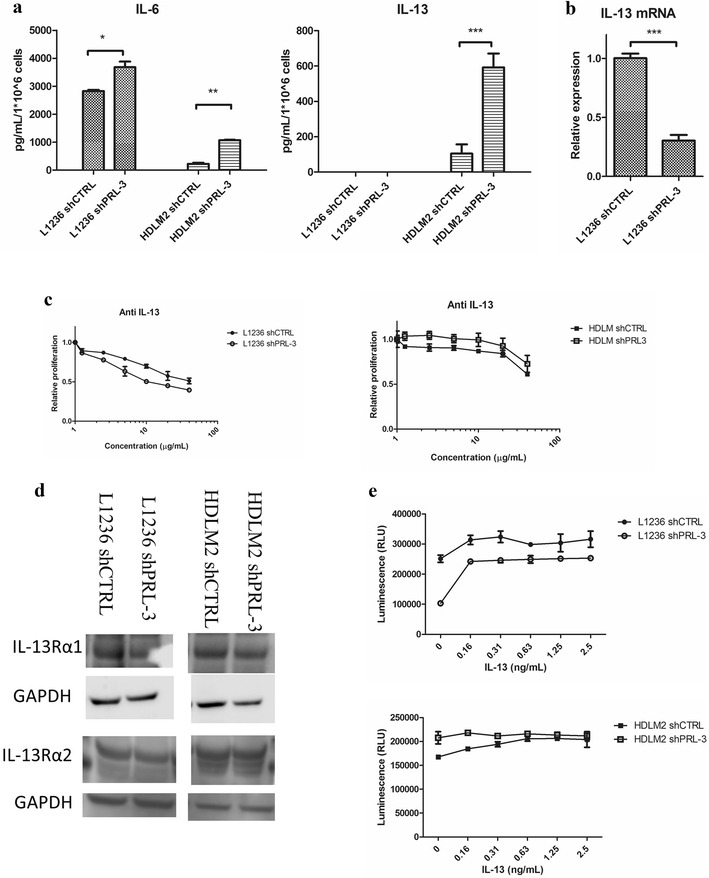
PRL-3 regulated cytokine production in cHL cell lines. **a** IL-6 and IL-13 secretion and **b** *IL*-*13* mRNA expression. Bars represent secreted cytokine detected by ELISA or *IL*-*13* expression measured by qRT-PCR. Figure shows one representative of three independent experiments. Error bars represent + 1 SD of duplicates. *p < 0.05. **p < 0.01. ***p < 0.001. **c** Neutralizing antibody against IL-13 for 48 h reduced proliferation as measured by CellTiter-Glo^®^ assay. Error bars represent ± 1 SD of triplicates. Figure shows one representative out of three independent experiments. **c** Western blotting of IL-13 receptor subunits alpha 1 and alpha 2. **d** Proliferation after stimulation with IL-13 for 48 h, as measured by CellTiter-Glo^®^ assay. Error bars represent ± 1 SD of triplicates. Figure shows one representative of three independent experiments

First, we quantified secreted IL-6 and IL-13. There was a 1.3- and 4.7-fold increase in secretion of IL-6 in L1236 shPRL-3 and HDLM2 shPRL-3, respectively, and a 5.7-fold increase in IL-13 secretion in HDLM2 shPRL-3 cells compared to control (Fig. 5a). We did not detect IL-13 secretion in either L1236 shCTRL or shPRL-3 by ELISA (Fig. 5a). However, by qRT-PCR we detected *IL*-*13* mRNA production, which was downregulated in L1236 shPRL-3 (Fig. 5b). Blocking IL-6 with neutralizing antibodies gave no significant difference in proliferation between PRL-3 knockdown or control in either L1236 or HDLM2 (data not shown). Neutralizing antibody against IL-13 reduced proliferation in L1236 shCTRL and shPRL-3, with 49 and 61% respectively (Fig. 5c). In HDLM2, there was 39% reduction in shCTRL and 27% in shPRL-3 (Fig. 5c). PRL-3 knockdown did not affect expression of IL-13 receptor subunits alpha-1 and -2 (Fig. 5d).

Next, we stimulated cells with IL-13 and examined proliferation. Interestingly, IL-13 increased proliferation of L1236 shPRL-3 with 245% (2.5 ng/mL), and 29% in L1236 shCTRL (Fig. 5e). Proliferation of HDLM2 shPRL-3 did not respond to IL-13 stimulation, while HDLM2 shCTRL increased proliferation with 22% (Fig. 5e). Taken together, this indicates that PRL-3 regulates secretion of proliferative cytokines, and in the case of IL-6 and IL-13, by lowering the production or secretion.

### PRL-3 enhanced STAT6 signaling

**Fig. 6:**
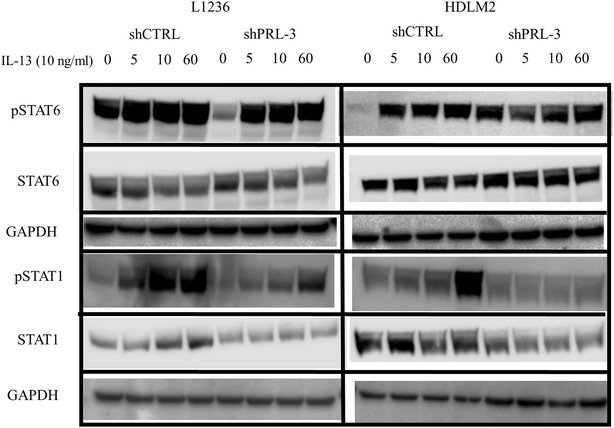
PRL-3 enhanced STAT6 signaling. Western blotting of pSTAT6, total STAT6, pSTAT1 and total STAT1 in L1236 and HDLM2 shCTRL and shPRL-3 stimulated with IL-13 (10 ng/mL) as indicated. Blots shows a representative blot of three independent experiments. Membranes were stripped and reprobed for total STAT6 or STAT1 and GAPDH

It has previously been shown that IL-13 activates the transcription factor STAT6 in cHL cell lines by phosphorylation. Thus, we investigated whether PRL-3 influenced activation of STAT6. L1236 shCTRL had constitutive phosphorylated STAT6 (pSTAT6), which was weaker in L1236 shPRL-3 (Fig. 6). Stimulation with IL-13 gave an increase in pSTAT6 in both. HDLM2 shCTRL had weaker constitutive pSTAT6 compared to HDLM2 shPRL-3 (Fig. 6). Stimulation with IL-13 gave an increase in pSTAT6 in HDLM2 shCTRL, but not in shPRL-3. For STAT1, PRL-3 expression led to increased phosphorylation after IL-13 stimulation in both L1236 and HDLM2 shCTRL (Fig. 6). There was a weak phosphorylation of STAT1 in L1236 shPRL-3 after 60 min, but we observed no increase in pSTAT1 in HDLM2 shPRL-3. Total STAT1 and STAT6 were unaffected by PRL-3 knockdown (Fig. 6).

## Discussion

With this study, we aimed to explore potential roles of PRL-3 in the pathogenesis of cHL. We show that PRL-3 protein was detected in a subset of lymph node biopsies from cHL patients. In microdissected HRS cells, *PRL*-*3* was overexpressed compared to normal control cells. Further, we show that inhibition of PRL-3 by shRNA or by small molecular inhibitors influenced cHL cell survival, reduced Mcl-1 expression and inhibited migration. PRL-3 knockdown also influenced IL-6 and IL13 secretion, and enhanced STAT6 signaling.

In our patient cohort, PRL-3 protein expression was detected in 16% of the samples. In a study by Wang et al., PRL-3 was elevated in an average of 22.3% (range 8.3–37.7%) of various cancer samples [23]. However, hematological cancers were not included in this work. High PRL-3 expression in hematological cancers has previously been shown by us and others [10, 12, 24]. In cHL, PRL-3 was introduced as a possible oncogene by Schwering et al. [15] based on differentially expressed genes in L1236 compared to germinal center B cells. In the same study, they confirmed *PRL*-*3* expression in three other cHL cell lines, including HDLM2. However, they did not compare expression in these cell lines with normal germinal center B cells. Consequently, they were not able to conclude whether *PRL*-*3* was overexpressed in cHL in general or only in L1236. In the present study, by using open source gene expression profiling data, we show that *PRL*-*3* is overexpressed in microdissected HRS cells, proving overexpression of PRL-3 in cHL. High PRL-3 expression is associated with metastasis in solid tumors, and poor prognosis in both solid and hematological cancers [25–27]. However, high PRL-3 expression in cHL did not correlate with clinical parameters, such as stage or survival, in either Steidl et al. [22], or in our cohort. In the study by Steidl et al. the HRS cells were not microdissected. To conclude whether PRL-3 is associated with negative prognosis, analysis from microdissected HRS cells should be done in a larger patient population. cHL is a disease with 86% survival for adults [4], and > 97% for children and adolescents [3]. Thus, finding genes associated with prognostic outcome, without a large number of samples, is difficult.

To explore cell survival effects of PRL-3, we transduced the cHL cell lines L1236 and HDLM2 with stable shRNA, or used small molecular inhibitors, against PRL-3. We found that inhibition of PRL-3 reduced proliferation and survival of L1236 and HDLM2 cells with the notable exception of knockdown in HDLM2. Knockdown of PRL-3 in L1236 reduced the expression of Mcl-1 but not in HDLM2. This may be an explanation for decreased survival in L1236 shPRL-3, and why there was no difference in survival by PRL-3 knockdown in HDLM2. We have previously shown that Mcl-1 expression in MM correlates with PRL-3 expression [11, 18]. Zhou et al. [24] found increased Mcl-1 expression in AML cell lines with forced expression of PRL-3. Nagel et al. [7] showed that Mcl-1 was highly expressed in HL cell lines, and induced by IL-6 expression as in other malignancies as well [7, 18].

Migration of cancer cells is important for dissemination of the cancer disease. In cHL, spread of tumor cells follows a typical pattern by first involving anatomically neighboring lymph nodes before dissemination to non-adjacent sites and organs [28]. Factors regulating spread of cHL cells are poorly understood [29]. In a study by Linke et al. [30] they show that cHL cell lines are highly migratory to a CCL19 gradient by RhoA and ROCK activation. In the present study, we show that knockdown of PRL-3 in L1236 and HDLM2 reduces migration towards a CCL19 gradient. Migration and motility have been linked to PRL-3 expression in several cancers [10, 12, 25, 26, 31, 32]. PRL-3 is associated with both RhoA and ROCK activation [13]. Taken together this indicates that PRL-3 could be important for cHL cells ability to disseminate.

**Fig. 7:**
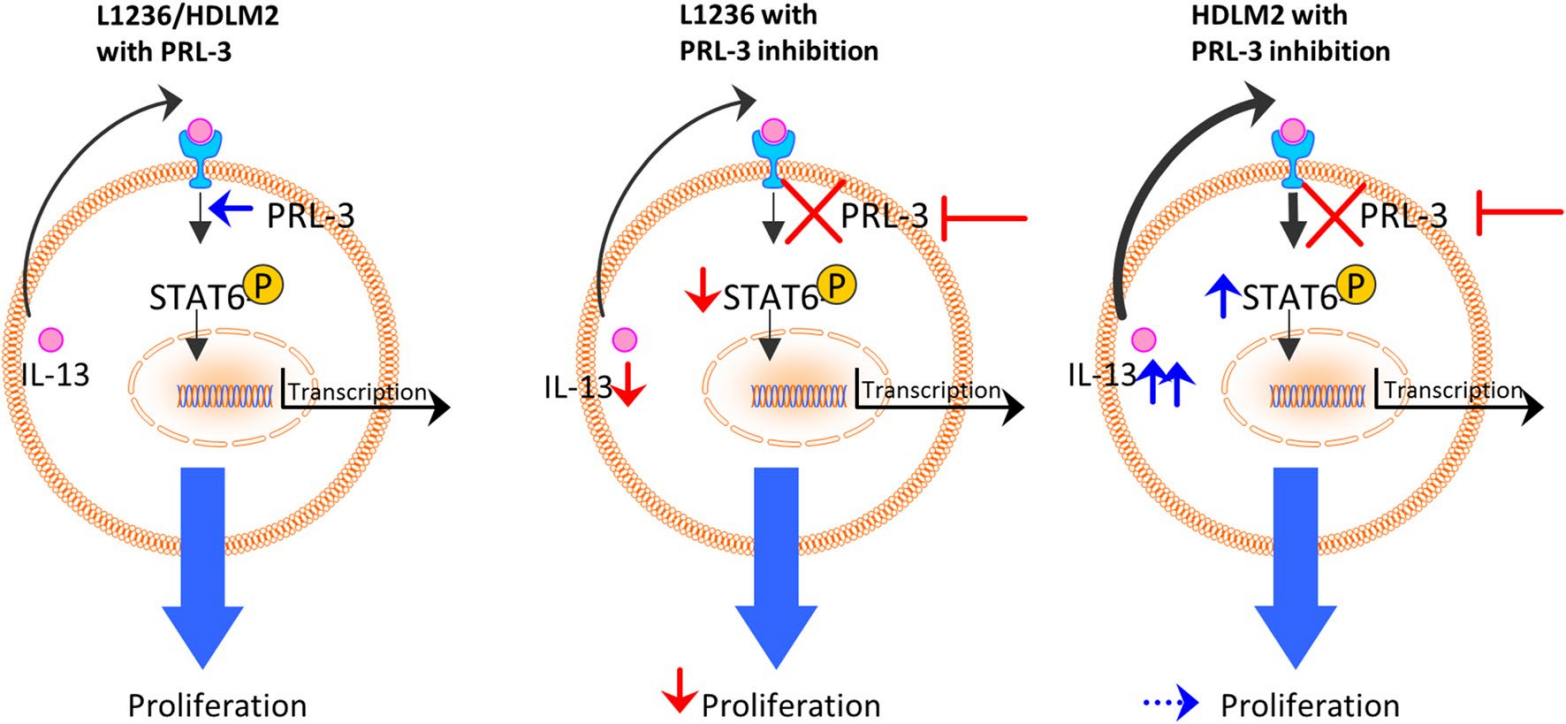
Suggested mechanism for a possible interplay between PRL-3, IL-13 and STAT6. With PRL-3 present (left), autocrine stimulation of IL-13 activates STAT6 and proliferation. With blocking of PRL-3 (right), STAT6 activity is reduced. L1236 cells do not possess the ability to increase IL-13 secretion and proliferation is reduced. HDLM2 cells can increase IL-13 secretion followed by an increase of pSTAT6 and thereby maintain cell growth

cHL cells are known to secrete cytokines that regulate cell behavior by autocrine or paracrine stimulation of cHL cells or neighboring cells in the microenvironment [8]. Constitutive STAT activation is a common feature in cHL [33, 34]. In cHL, STAT6 activation is mainly IL-13-driven, and IL-13 is an important growth factor in cHL [34]. Both HDLM2 and L1236 secrete IL-13, but detection of IL-13 secretion by ELISA in L1236 has been challenging because of low levels [34, 35]. We detected *IL*-*13* mRNA in L1236, but not protein by ELISA. PRL-3 has previously not been associated with STAT6 signaling. There are, however, several studies linking PRL-3 expression to STAT1, STAT3 and STAT5 activation [18, 25, 36]. STAT3 and STAT5 are transcriptional regulators of PRL-3 in AML cells [25, 36]. We have previously shown that PRL-3 expression can lead to constitutive activation of STAT3 in MM cells [18]. Our present results show that PRL-3 knockdown alter STAT6 activation. Knockdown of PRL-3 reduced constitutive pSTAT6 in L1236, whereas for HDLM2 there was an increase in pSTAT6. The latter probably happened because HDLM2 responded to PRL-3 knockdown by increasing IL-13 secretion. L1236 cells, on the other hand, did not respond this way to PRL-3 knockdown. To the contrary, reduced amount of IL-13 mRNA expression, and loss of constitutive pSTAT6, indicate that PRL-3 induces survival in L1236 cells via an autocrine IL-13 loop, although no IL-13 protein could be detected in the supernatant. A possible mechanism is suggested in Fig. 7. Increased sensitivity to anti-IL-13 by L1236 shPRL-3 cells compared to control cells in a proliferation assay supports this assumption.

A link between PRL-3 and IL-13-driven proliferation is further supported by our results from IL-13 stimulation. IL-13 increased proliferation by 245% in L1236 shPRL-3. In a work by Skinnider et al., L1236 cells were very sensitive to IL-13 neutralization and stimulation, through regulation of STAT6 activation [34]. IL-13 stimulation increased proliferation of HDLM2 shCTRL, but not shPRL-3. The lack of increase in HDLM2 shPRL-3 is probably due to saturation with IL-13 in an autocrine loop. Kapp et al. [8] did not see any increase in proliferation by IL-13 stimulation in HDLM2, but they used higher concentrations and a different method. The results from the small molecular inhibitors further support that PRL-3 is important for survival of cHL cells. The cells were most sensitive towards thienopyridone, a PRL family inhibitor. Thienopyridone, and its derivate iminothienopyridinedione 13, are known as the most potent PRL-3 inhibitors [37–40]. In MM and gastric cancer cell lines, sensitivity to PRL-3 inhibitor I was not related to the PRL-3 level [18, 41], as we also show here for L1236 and HDLM2. Taken together, our data suggest that PRL-3 influences survival of HL cells, probably through regulation of IL-13 secretion, phosphorylation of STAT6 and expression of Mcl-1.

## Conclusion

In this study, we show that PRL-3 protein was expressed in 16% of cHL patients and that PRL-3 was overexpressed in HRS cells compared to normal controls. Inhibition of PRL-3 influenced cytokine production and reduced migration, proliferation and viability, probably through regulating Mcl-1 expression and IL-13-stimulated activation of STAT6.

## Additional files

**Additional file 1. Supplemental Figure S1:** IHC of normal lymph node and prostate cancer (postive control)

**Additional file 2. Supplemental Figure S2:** PBMC from healthy volunteers treated with PRL-3 inhibitors

## Abbreviations

PRL-3: phosphatase of regenerating liver-3
HL: Hodgkin lymphoma
NHL: non-Hodgkin lymphoma
cHL: classical Hodgkin lymphoma
HRS: Hodgkin and Reed–Sternberg
NF-κB: nuclear factor κB
JAK: janus kinase
STAT: signal transducer and activator of transcription
NK: natural killer
IL: interleukin
MM: multiple myeloma
ALL: acute lymphoblastic leukemia
PBMC: peripheral blood mononuclear cells
Mcl-1: myeloid cell leukemia-1
IHC: immunohistochemistry
qRT-PCR: quantitative real time PCR
PI: propidium iodide
CCL: C-C motif chemokine ligand
GM-CSF: granulocyte–macrophage colony-stimulating factor
CXCL: C-X-C motif chemokine ligand
VEGF: vascular endothelial growth factor
