# Supplementary material for: Phosphatase of regenerating liver-3 (PRL-3) is overexpressed in classical Hodgkin lymphoma and promotes survival and migration
Source: Exp Hematol Oncol. 2018 Apr 10;7:8. doi: 10.1186/s40164-018-0100-2 (PMC5894150; doi:10.1186/s40164-018-0100-2)
Supplement: Supplementary file 1 — Additional file 1. Supplemental Figure S1: IHC of normal lymph node and prostate cancer (postive control) [file 40164_2018_100_MOESM1_ESM.docx]

Additional file 1


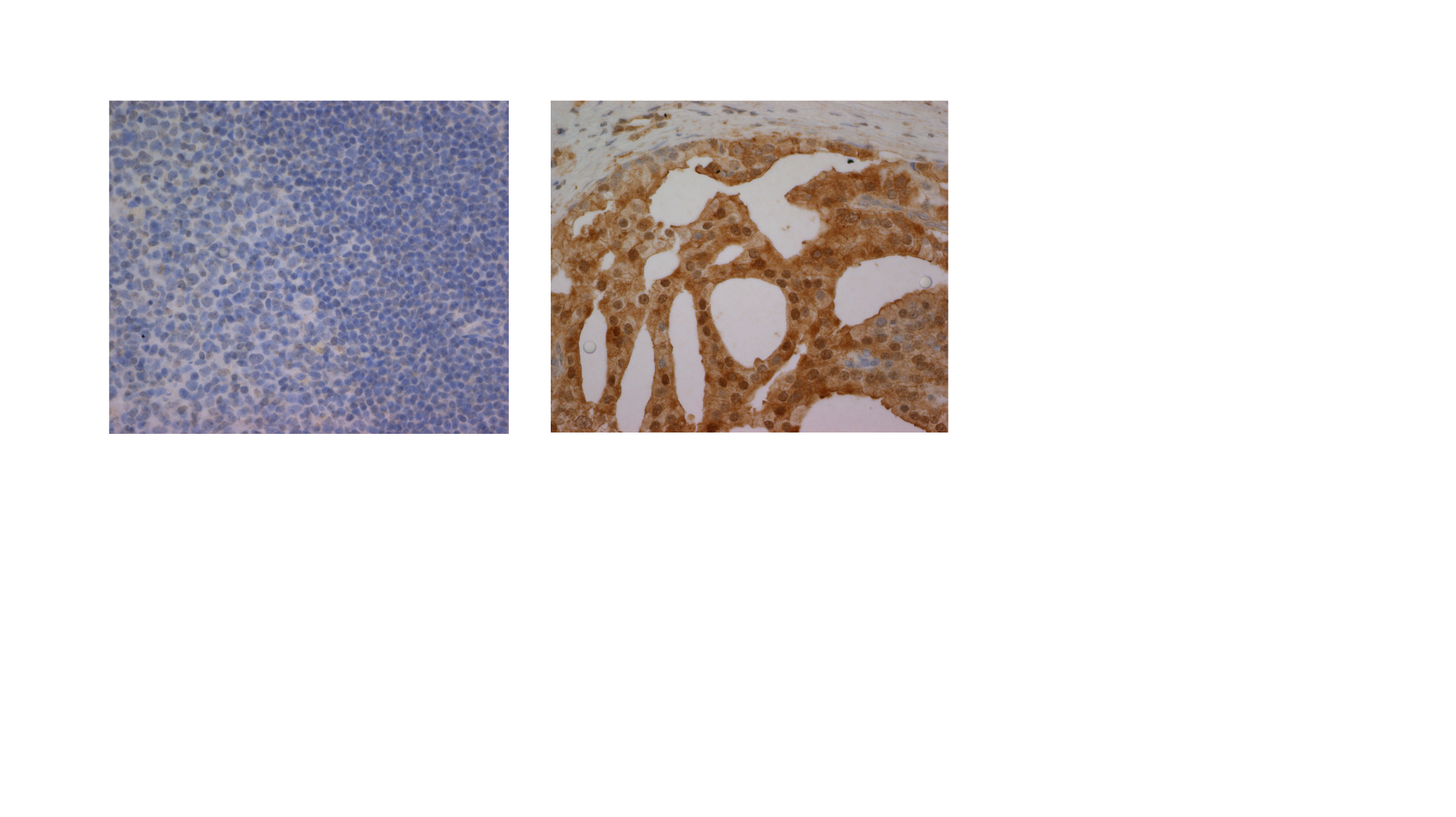


Cells in a normal lymph node (left) do not express PRL-3 (negative control). Prostate cancer cells (right) is highly positive for PRL-3 expression (positive control). Microscopic images were obtained with a Lumenera Infinity 2 camera and Infinity analyze software, release 6.2 (Lumenera Corporation, Ottawa, Ontario, Canada) using a Nikon eclipse Ci microscope (Nikon Gmbh, Düsseldorf, Germany) at 400x magnification.
