# Supplementary material for: Phosphatase of regenerating liver-3 (PRL-3) is overexpressed in classical Hodgkin lymphoma and promotes survival and migration
Source: Exp Hematol Oncol. 2018 Apr 10;7:8. doi: 10.1186/s40164-018-0100-2 (PMC5894150; doi:10.1186/s40164-018-0100-2)
Supplement: Supplementary file 2 — Additional file 2. Supplemental Figure S2: PBMC from healthy volunteers treated with PRL-3 inhibitors [file 40164_2018_100_MOESM2_ESM.docx]

Additional file 2


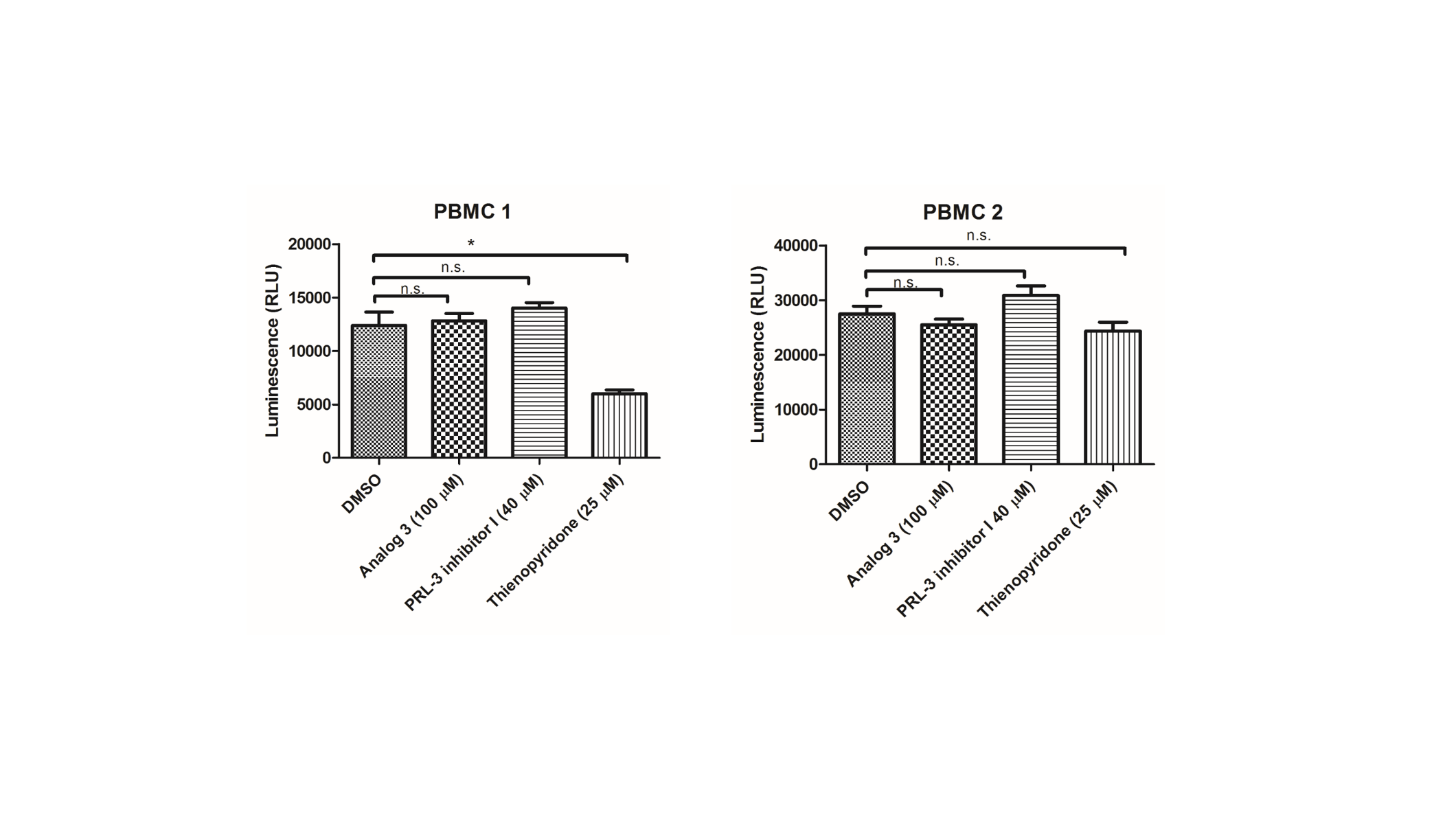


PBMC isolated from two healthy volunteers were treated with three different PRL-3 inhibitors and DMSO as control. Proliferation was measured by CellTiter-Glo® assay. *Error bars* represent + 1 SD of triplicates. * indicates p < 0,05. n.s. indicates not significant.
